# Empowering Mimicry: Female Leader Role Models Empower Women in Leadership Tasks Through Body Posture Mimicry

**DOI:** 10.1007/s11199-018-0911-y

**Published:** 2018-04-07

**Authors:** Ioana M. Latu, Marianne Schmid Mast, Dario Bombari, Joris Lammers, Crystal L. Hoyt

**Affiliations:** 1 Center for Identity and Intergroup Relations, Queen’s University Belfast, 18-30 Malone Road, Belfast, BT9 5BN UK; 2 Faculty of Business and Economics, University of Lausanne, Lausanne, Switzerland; 3 Social Cognition Center Cologne, University of Cologne, Cologne, Germany; 4 Jepson School of Leadership Studies, University of Richmond, Richmond, VA USA

**Keywords:** Leadership, Role models, Nonverbal communication, Imitation, Empowerment, Human females, Gender

## Abstract

In two studies we investigated the behavioral process through which visible female leader role models empower women in leadership tasks. We proposed that women tend to mimic the powerful (open) body postures of successful female role models, thus leading to more empowered behavior and better performance on a challenging leadership task, a process we called *empowering mimicry*. In Study 1, we experimentally manipulated the body posture of the male and female role models and showed that 86 Swiss college women mimicked the body posture of the female (ingroup) but not the male (outgroup) role model, thus leading to more empowered behavior and better performance on a public speaking task. In Study 2, we investigated the boundary conditions of this process and showed that empowering mimicry does not extend to exposures to non-famous female models among 50 Swiss college women. These findings suggest that nonverbal mimicry is one important mechanism through which female leader role models inspire women performing a challenging leadership task. From a practice perspective, our research underscores the importance of female leaders’ visibility because visibility can drive other women’s advancement in leadership by affording women the opportunity to mimic and be empowered by successful female role models.

**Electronic supplementary material:**

The online version of this article (10.1007/s11199-018-0911-y) contains supplementary material, which is available to authorized users.

Female role models can inspire girls and women in male-typical domains such as STEM (Cheryan et al. 2011; Dennehy and Dasgupta 2017), athletics (Greendorfer 1977), and managerial and political leadership (Latu et al. 2013; Simon and Hoyt 2013; Singh et al. 2006; Wolbrecht and Campbell 2007). There are several mechanisms that can account for these positive effects, including women’s increased sense of belonging and self-confidence (Dennehy and Dasgupta 2017). In the current study, we propose an alternative, behavioral mechanism that explains how political female role models inspire women in leadership tasks.

Specifically, we suggest that visible female role models in leadership may offer the opportunity for modeling empowering behaviors in women faced with leadership challenges. Essentially, female leader role models can show women how to behave in challenging situations—how to speak, stand, or move. In turn, women may model those behaviors and, as a result, be empowered by the female leader role models. More precisely, in the context of the current studies we propose that when women are exposed to visible female leader role models, they are likely to imitate those role models’ nonverbal behavior, which ultimately empowers women during leadership challenges—a process we call *empowering mimicry*. As such, we argue that increasing women’s visibility in leadership is important because female leaders’ visibility is the engine that can further drive the advancement of women in leadership, by offering women the opportunity to imitate and be empowered by successful female role models.

We focus on political leadership for two reasons. First, the current political context affords increased visibility for female politicians (e.g., Angela Merkel in Germany, Theresa May in the UK, Hillary Clinton in the U.S.), and we believe it is important to investigate how such visibility affects women. Second, political leadership tasks (e.g., giving speeches) are ideal tasks for measuring both nonverbal behavior and performance and for obtaining quantitative measures of mimicry and empowerment. However, although we empirically investigate political leadership, we would expect our findings to extend to other leadership domains.

## Female Role Models in Leadership

The effects of highly successful female role models on women in leadership are mixed, suggesting that female role models have the potential of having both deflating and inspiring effects. For example, some research suggested that women who are exposed to highly successful women may think that they can never achieve the same level of success and, as a result, feel discouraged. Indeed, exposures to female role models led to lower self-evaluations and leadership aspirations (Hoyt and Simon 2011), lower self-ratings of competence (Parks-Stamm et al. 2008), and lower self-leadership associations (Rudman and Phelan 2010).

However, the bulk of the existing work suggests that exposures to female leader role models can lead to positive outcomes for women. Several studies have shown that successful women can be inspiring in demonstrating that success is attainable. In one line of research, both experimental exposure and long-term quality interactions with female leaders predicted stronger implicit self-concept of leadership and stronger career ambitions (Asgari et al. 2010; Asgari et al. 2012; Dasgupta and Asgari 2004). Similarly, Simon and Hoyt (2013) found that women exposed to media images of women depicted in counter-stereotypical roles reported stronger nontraditional gender role beliefs, less negative self-perceptions, and greater leadership aspirations than did women exposed to images of women in stereotypical roles.

Specifically looking at behavioral outcomes, Latu et al. (2013) showed that subtle exposures to a picture of an elite female leader positively influenced women’s leadership behavior and self-appraisals. In a stressful leadership task in which participants gave a persuasive speech in front of an audience, women showed more empowered behavior (operationalized by longer speeches) and better speech performance (coded by an external rater) when exposed to a female leader role model (Hillary Clinton or Angela Merkel) compared to a male role model (Bill Clinton) or no role model at all. Furthermore, such effects only occurred for women and not for men.

We are interested in the behavioral mechanism of this effect: How do female leader role models empower women’s behavior in a leadership task? From a psychological perspective, this can be the case because a visible role model affords the opportunity for mimicry*.* In fact, with some exceptions, one difference between the studies finding contradictory effects was that those studies that found inspiring effects tended to have more visible role models—either in the form of images of women presented during the task (Latu et al. 2013) or as naturalistic interactions with women (Asgari et al. 2010). This observation led us to hypothesize that the actual visibility of female role models may be vital to producing empowering effects. In other words, visible female leader role models may literally show women how to be and act in certain situations. Specific to leadership tasks, women may mimic the actual powerful nonverbal behaviors of the model (e.g., powerful body postures), which could, in turn, lead to more empowered behaviors and enhanced performance. We call this two-step process *empowering mimicry.* The steps of this process are described in detail in the following.

## Behavioral Mimicry

Individuals tend to sync and mimic each other’s facial expressions (Blairy et al. 1999; Dimberg et al. 2000), body postures (Bernieri 1988; LaFrance and Broadbent 1976), gestures (Chartrand and Bargh 1999; Yabar et al. 2006), and speech accents and patterns (Cappella and Planalp 1981; Giles and Powesland 1975; Webb 1969). This phenomenon is called behavior matching (Bernieri 1988) or nonconsious mimicry, and it tends to occur outside our awareness (Chartrand and Bargh 1999).

Initially, the goal of mimicry was thought to be affiliation: People mimic each other as a way of increasing liking for the interaction partner (chameleon effect; Chartrand and Bargh 1999) and building harmonious social interactions (Vacharkulksemsuk and Fredrickson 2012). More recently, it was suggested that mimicry is also driven by a learning goal, such that individuals mimic others in order to produce the appropriate response to a situation (Hess and Fischer 2017; Kavanagh and Winkielman 2016). In other words, we mimic others in order to learn how to act and react in a given situation. Thus, if the goal of mimicry is learning how to behave, mimicry of ingroup members is preferred because ingroup members’ signals are seen as more adaptive and trustworthy compared to outgroup members’ signals. Supporting this argument, individuals are more likely to mimic ingroup rather than outgroup members (Bourgeois and Hess 2008; Lakin et al. 2008; van Baaren et al. 2009; Yabar et al. 2006).

Consistent with this learning view of mimicry, we propose that women will mimic female role models during a novel, stressful task, given that learning the nonverbal signals produced by these successful role models may help women respond appropriately to the stressful situation. More precisely, women will adopt more powerful, dominant postures as a result of imitating the powerful female role models. Importantly, we propose that women will only mimic female, but not male, role models because of ingroup mimicry effects. We argue that gender is a relevant ingroup-outgroup dimension given that the context (i.e., nature of the task, cover story, political role models) primes political leadership, which is stereotypically associated with masculinity (Koenig et al. 2011) and can induce stereotype threat (Davies et al. 2005; Gupta and Bhawe 2007; Von Hippel et al. 2011).

Two clarifications are needed. First, the term mimicry is most often used to denote imitation of dynamic behaviors in social interactions. However, mimicry of static models can also occur, although effects tend to be smaller. For example, participants mimicked both static and dynamic emotional facial expressions, although mimicry was enhanced by exposures to dynamic expressions (Rymarczyk et al. 2011; Rymarczyk et al. 2016; Weyers et al. 2006). Moreover, a similar procedure of having participants mimic a body posture was successfully used and led to empowered behavior (Arnette and Pettijohn 2012). Based on this evidence, we will use the term *mimicry* for imitation of body postures of static role models.

Second, an alternative to mimicry (i.e., complementarity) is also a possible response. Tiedens and Fragale (2003) showed complementarity responses, such that participants decreased their postural openness when exposed to confederates with open postures while interacting during a cooperative task. This complementary response is motivated by a desire to maintain existing hierarchies during cooperative interactions. However, there is relatively little research regarding complementarity versus mimicry responses, and some research suggests the effect is moderated by situational factors (e.g., complementarity responses occurred when the interaction partner was smiling, but not when he was not; de Lemus et al. 2012). Furthermore, if behavioral responses depend on a person’s motivations and goals, complementarity is less likely to occur if individuals are not cooperating and are not motivated to maintain an actual hierarchy. As such, we posit that mimicry is more likely to happen when exposed to iconic female role models, given that the goal is to learn how to behave in a novel, stressful situation rather than to interact with a person. It is only in the latter case that a hierarchy would readily emerge among social interaction partners, accounting for the complementary behaviors. In summary, we propose that women performing a challenging leadership task will be motivated to mimic female, but not male, leadership role models because mimicking the highly successful female role model’s nonverbal behavior would help them produce the appropriate, successful response in the situation.

## Performance Effects

We further propose that the change in women’s body posture will affect women’s empowered behavior and performance in a leadership task. Indeed, recent experimental research suggests that adopting open, expansive body postures can increase people’s power-related cognitions and behaviors given the right contextual factors. Compared to participants who held closed, restricted body postures, participants who held open, expansive body postures subsequently showed increases in self-reported feelings of power (Carney et al. 2015; Fischer et al. 2011; Park et al. 2013; Ranehill et al. 2015), as well as more risky, reward-oriented decisions in a gambling task (Carney et al. 2010). Moreover, adopting open power poses before a self-presentation task improved performance on the task (Cuddy et al. 2015). Finally, participants who adopted an erect (vs. slouched) body posture chose seats closer to the head of the table, indicating empowered leadership behavior (Arnette and Pettijohn 2012).

It should be noted that the evidence for the effect of open body postures on power-related outcomes is mixed. For example, using a high-powered sample, Ranehill et al. (2015) did not replicate Carney et al.’ (2015) findings on risk-taking. However, one consistent effect that has emerged is that postural openness increases subjective feelings of power (see Gronau et al. 2017, for a recent Bayesian meta-analysis of six multi-lab, pre-registered studies; Carney et al. 2015, for a qualitative review of the literature). The effect of open postures on subjective outcomes was also supported by a recent p-curve analysis of 55 studies (Cuddy et al. in press).

This subjective outcome, which has received strong empirical support, is the significant one for our research given that feeling powerful has a multitude of positive consequences: Consistent with the approach/inhibition theory of power, feeling powerful enhances cognitive abilities and goal-oriented behaviors (Guinote 2007), and it increases positive mood and reduces fear of negative evaluations (Keltner et al. 2003). Supporting these effects in a social-evaluation situation, participants who thought about having power over other people (a common power manipulation) before giving a stressful self-presentation task felt less fear of negative evaluation and performed better on the self-presentation task (Schmid and Schmid Mast 2013).

Consistent with this evidence, we propose that women will perform better on a leadership task as a result of holding more open body postures. However, unlike previous studies in which such empowering effects were obtained by explicitly asking participants to adopt open body postures for several minutes (Cuddy et al. 2015) or to think about situations in which they felt powerful (Schmid and Schmid Mast 2013), in our studies we propose that such empowering effects may arise from imitating successful female leader role models.

## Summary and Overview

We propose and test a two-step process we call empowering mimicry. As a first step of the process, consistent with the literature on unconscious mimicry (Chartrand and Bargh 1999) and the proposition that individuals mimic in order to learn appropriate responses (Hess and Fischer 2017; Kavanagh and Winkielman 2016), we propose that women mimic the body posture of female leader role models. If the role model shows an open body posture, women will mimic this posture during a leadership task by showing more open postures themselves. Importantly we propose that women will only mimic the female, but not the male, role model because of ingroup effects (Yabar et al. 2006). In a second step, the mimicked body posture will affect performance, consistent with the literature that shows that individuals who adopt open postures feel and behave in a more empowered way (Carney et al. 2015). When women mimic an open body posture, they behave in a more empowered way and perform better on a leadership task. The purposes of our empirical investigation are both to understand the process through which female leader role models can be empowering and to underscore the importance of women’s visibility in leadership.

We only focused on female participants for two reasons. First, previous work suggested that women, but not men, were empowered by same-gender role models (Latu et al. 2013). In fact, men’s empowered behavior remained constant and high when exposed to male, female, or no role models. As such, there was no role model effect that would need to be explained. A second, related reason is that, within leadership, women are the ones affected by stereotype threat (Davies et al. 2005; Gupta and Bhawe 2007; Von Hippel et al. 2011) and most in need of empowering interventions.

We designed two behavioral studies to test the empowering mimicry process and its boundary conditions. If empowering mimicry is one process that underlines the inspiring effects of female leader role models, we should see that women change their body posture when exposed to the female leader role models and that this posture change mediates the effect of role models on performance. Moreover, women should only mimic female but not male role models because of ingroup effects (Bourgeois and Hess 2008; van Baaren et al. 2009; Yabar et al. 2006). These predictions were tested in the first study in which we experimentally manipulated the body posture (open vs. closed) of female and male role models.

In Study 2, we further explored the boundary conditions of the empowering mimicry process by investigating under what conditions mimicry occurs. Does mimicry depend on exposure to a clearly identified female leader or are women likely to mimic any women to whom they are exposed? To answer this question, we investigated whether women would mimic and be empowered by an unknown female role model. This question is theoretically important because open body postures may elicit complementary rather than mimicry effects in regular social interactions, as we previously discussed (Tiedens and Fragale 2003). This tendency to complement is believed to serve the goal of maintaining power/dominance hierarchies within social interactions. It is possible that an unknown woman is not perceived as a successful role model from whom participants could learn how to behave successfully. If, instead, the model is seen as a possible interaction partner, it could be that women would complement rather than mimic an unknown woman who is not seen as a clear leadership icon. This finding would underline the importance of having clearly identified, iconic female role models in leadership.

In both studies we used a behavioral paradigm and measured participants’ actual nonverbal behavior and performance in a simulated leadership task. Participants’ task was to deliver a persuasive speech in front of a 12-person avatar audience in an Immersive Virtual Reality Environment. Giving a public speech in front of an audience is a task typical of many leadership positions, and it requires communication and persuasive skills necessary for successful leadership. Also, giving a speech in a virtual environment typically elicits high levels of stress similar to giving a public speech in a real environment (Pertaub et al. 2001). In fact, virtual reality is such a powerful platform for inducing naturalistic levels of stress when giving presentations that it has been used in behavioral therapies for treating public-speaking anxiety (Anderson et al. 2005). For the purpose of our study, the virtual reality technology had the advantage of allowing us to study actual behavior in an experimentally controlled environment in which we standardized the behavior and reactions of the avatar audience. This set-up also allowed us to expose participants to female and male role models during the task by hanging a picture of the role model on the virtual wall participants were facing.

Given that participants delivered actual speeches, across both studies we were able to measure several concrete behaviors based on video/audiotapes of the speeches. We measured body posture openness, speaking time, and actual speech performance evaluated by external raters. For each measure we used two independent coders who were unaware of the study’s hypotheses and experimental conditions. We also measured speaking time because it is a measure of power and dominance (Schmid Mast 2002) whereby powerful people tend to speak longer. Moreover, the longer people speak, the more influential they are (Chaiken et al. 1989). Speaking time also has the advantage of being an objective measure that bypasses evaluators’ biases.

We measured speech performance using two methods, both of which consist of two trained coders who rated performance based on speech recordings. As a first measure, we were interested in the overall speech performance rated based on all available cues, both visual and audio. This type of evaluation would closely mimic the evaluations speakers would receive in a real-life situation. As a second measure of performance, two coders assessed speech performance based on an audio recording only. We chose this coding strategy in order to prevent a halo effect in which speeches were rated better because the participants displayed open body postures, which usually signal power and dominance (Hall et al. 2005). As such, if we also observe effects on the audio-rated speech, we can assert that there is a true effect of role models through posture on speech quality rather than merely a halo effect.

## Study 1

In our first study we experimentally investigated whether changes in the body posture of a female leader role model can influence the body posture of the women exposed to the model and can subsequently influence behavior and performance. As such we manipulated the expansive versus restricted body posture of one known female (Hillary Clinton) and one known male role model (Bill Clinton). We specifically investigated whether female college students would mimic the female role model and whether this change in posture is, at least in part, responsible for differences in performance of women performing leadership tasks.

We proposed two hypotheses. First, we hypothesized that women would mimic the body posture of the female, but not the male, role model such that they would display significantly more open body postures when exposed to the female leader role model with an open body posture compared to the female leader role model with a closed body posture. Second, we hypothesized a moderated mediation whereby women’s increases in postural openness in response to the role model would account for their enhanced performance, but only when the role model was female and not male. We expected this moderated mediation across all behavioral outcomes measured: empowered behavior (i.e., speaking time) and two assessments of rated performance.

We also measured two control variables that could be related to our outcomes. First, consistent with previous research using virtual reality technology (Bombari et al. 2015; Price et al. 2011), we assessed the degree to which participants subjectively perceived the virtual environment as being “real” while completing the task. Second, we measured participants’ age because older participants may be more dominant and have more experience with public speaking.

### Method

#### Participants and Procedure

**Fig. 1:**
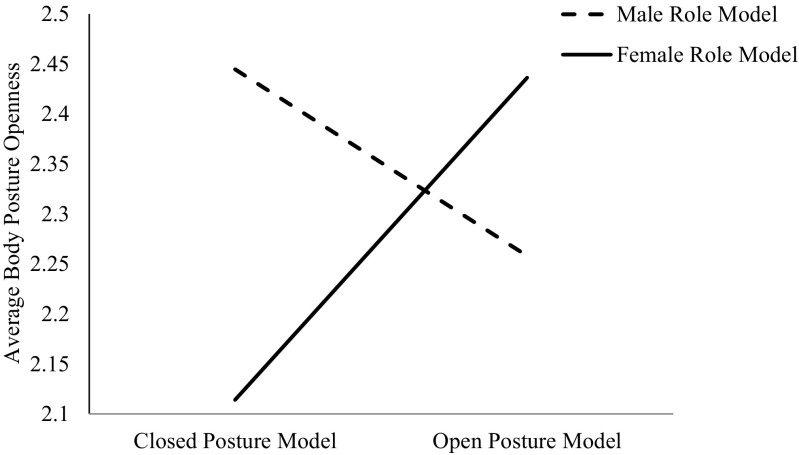
The interactive effects of role model gender and role model posture on body posture openness for female participants in Study 1

We recruited 86 female participants (*M*_age_ = 21-years-old, *SD* = 1.66, range = 18–24) at a Swiss University. Participants’ task was to give a persuasive political speech arguing against an increase in student fees. Participants composed their own speeches and they were given five minutes to prepare their main ideas. They were told to focus on creating a convincing speech that would be delivered to university administrators, in a room designed using Immersive Virtual Environment Technology (IVET). Participants wore a mobile head-mounted display through which they experienced a virtual room containing an audience of 12 avatars (half women, half men, presumably university administrators). The avatars were programmed to follow the participants’ movements with their eyes and heads. (A still image of the virtual room from the participant’s perspective can be seen in Figure 1s of the online supplement.)

As a cover story, we told participants that they were randomly assigned to one of the rooms of the university’s Political Science department in which a picture of a different famous politician was displayed in each room. Depending on the randomly assigned condition, a picture of a role model politician was shown hanging on the virtual wall opposite the participant. Participants were randomly assigned to one of four conditions: either a known female (U.S. Presidential candidate Hillary Clinton) or a known male role model (former U. S. President Bill Clinton) displaying either an open (expansive) or closed (restricted) body posture (see Figure 2 of the online supplement for these stimuli materials).

In a pretest, 27 participants rated pictures of the two politicians Hillary Clinton and Bill Clinton, on several dimensions. Independent *t*-tests revealed that participants did not perceive the two politicians to be different in terms of liking, *t*(25) = .62, *p* = .54, *d* = .24, charisma, *t*(25) = .51, *p* = .61, *d* = .19, competence, *t*(25) = 1.17, *p* = .25, *d* = .45, and perceived power, *t*(25) = 1.04, *p* = .31, *d* = .40. It should be noted that data were collected before the 2016 U.S. Presidential election (which included Hillary Clinton) and that the study’s participants were non-voting Swiss. This fact may explain participants’ similar views of Hillary and Bill Clinton because both targets were seen as familiar leadership icons, but were somewhat removed from personal political preferences.

#### Body Posture Manipulation

The open and closed body postures were obtained by superimposing the heads of Hillary Clinton and Bill Clinton on pictures of posed postures by a female and a male actor (the same head was used for both postures) using Photoshop (see Figure 2s in the online supplement). Before using these pictures, we pre-tested them to insure that they were perceived as powerful versus submissive postures. Thirty-two pilot Swiss college students (19 female), different from the ones in the main study, were assigned to rate the dominance of one target in a 2 (Target gender) × 2 (Target pose: open vs. closed) between-subjects design. Dominance was rated by indicating their agreement with one item (“I find this person to be dominant”) on a scale from 1 (*Strongly disagree*) to 7 (*Strongly disagree*). We also measured participants’ liking of the politician (“I like this person”) on a similar 7-point scale, given that raters’ own opinions and preferences for the politicians may influence their dominance ratings. A 2 × 2 between subjects ANCOVA, controlling for participants’ liking for the target, revealed a main effect of target pose, such that open posture targets (*M* = 4.54, *SD* = 1.59) were perceived as more dominant compared to closed posture targets (*M* = 3.70, *SD* = 1.83), *t (27)* = 2.26, *p* = .03, *d* = .49. Importantly, this effect was found regardless of the gender of the target: the interaction between target pose and target gender was not significant, *F*(1, 27) = .13, *p* = .72. In other words, both male and female open posture models were perceived as more dominant compared to closed posture models.

#### Body Posture Openness and Speaking Time

Two independent coders (unaware of the experimental conditions and study hypotheses) rated the openness of the body posture of the participants on a scale from 1 (*arms/legs close to body; very closed body posture*) to 5 (*arms/legs away from body; very open body posture*). We used the same coding strategy as Tiedens and Fragale (2003) who stopped the tape every minute to assess body posture openness. However, to obtain a more fine-grained measure, our coder stopped the video recording every 30s (starting at 10s, because the first few seconds usually involve the participants preparing and taking their speech positions) and evaluated the openness of the body posture at each of these points. For each participant, we averaged these scores to obtain a measure of overall body posture openness throughout the speech. The reliability between the two coders computed based on the entire sample was good: Krippendorff’s *α* = .85. We averaged the scores from both coders (*M* = 2.31, *SD* = .55, *range =* 1–3.44) and used them in further analyses. Speaking time was measured in seconds by the experimenter, using a chronometer, from the first to the last word uttered. We did not perform any transformations on the time data.

#### Speech Performance Video

Two coders evaluated overall speech quality based on videotapes, which allowed them to both see and hear the participant. The coders used the same coding scale as the one used in Latu et al. (2013). Speech performance was assessed on a scale from 1 (“*If somebody heard this speech, they would not be convinced at all*”) to 5 (“*If somebody heard this speech, they would be convinced*”). The reliability between the two coders computed based on the entire sample was good: Krippendorff’s *α* = .85. We averaged the scores from both coders (*M* = 2.41, *SD* = 1.00, range = 1–5) and used them in further analyses.

#### Speech Performance Audio and IVET Realness

Two coders assessed each audiotape in terms of overall speech quality defined as the degree to which the speech was persuasive. It involves having good, original, well-organized arguments and appropriate examples. It also takes into consideration the vocal quality of the presenter, such as a loud voice, normal speed, and appropriate emphasis on key words. Speech quality was assessed on a scale from 1 (“*If somebody heard this speech, they would not be convinced at all*”) to 5 (“*If somebody heard this speech, they would be convinced*”). Although the reliability between the two coders computed based on the entire sample was relatively low, Krippendorff’s *α* = .71, we accepted it given the highly subjective nature of the coded variable and because it will be interpreted in conjunction with other outcome variables which are either objectively measured (speaking time) or have high reliability (speech performance video). We averaged the scores from both coders (*M* = 2.95, *SD* = .80, range = 1–4.50) and used them in further analyses. To assess IVET realness, participants rated one item (“For me, the situation in the virtual world was hard to believe”) on a scale from 1 (*Strongly disagree)* to 5 (*Strongly Agree*).

### Results

#### Preliminary Data and Analysis Plan

Table 1 presents bivariate correlations, means, and standard deviations for all control and outcome variables. We first investigated whether our control variables were significantly correlated with our outcomes of interest (see Table 1). Whereas participants’ age was not significantly related to any of our outcomes, IVET realness was significantly correlated with several outcomes. Specifically, the less real the IVET environment felt to participants, the less open was their body posture, the shorter were their speeches, and the worse they performed. Because of these patterns, we included IVET realness as a control variable in further analyses.

**Table 1:** Descriptive statistics and correlations study variables, Study 1

			Correlations								
Variables	*M*	*SD*	1	2	3	4	5	6	7	8	9
1. Body posture openness 1	2.37	.60	–	.87**	.38**	.42**	.45**	.33**	.24*	−.01	−.28**
2. Body posture openness 2	2.25	.54		–	.40**	.43**	.42**	.31**	.24*	−.06	−.24*
3. Speaking time	147.54	62.10			–	.41**	.42**	.37**	.41**	−.06	−.22*
4. Speech performance video 1	2.51	1.06				–	.87**	.35**	.34**	.10	−.20
5. Speech performance video 2	2.30	1.02					–	.49**	.47**	.14	−.24*
6. Speech performance audio 1	3.01	.82						–	.70**	.10	−.09
7. Speech performance audio 2	2.90	.92							–	.13	−.27*
8. Age	20.75	1.66								–	−.01
9. IVET realness	1.75	1.06									–

*n* = 86. IVET = Immersive Virtual Environment Technology

*p < .05. **p < .01

To investigate our two hypotheses, we tested three moderated mediation models predicting empowered behavior (speaking time) and externally evaluated speech performance (coded video and audio), respectively. The models propose role model body posture openness as the predictor variable, participants’ body posture openness as the mediator, and the role model’s gender as the moderator of the predictor–mediator path. IVET realness was included as a control variable. The models were tested using Hayes’ PROCESS macro Model 7 (Hayes 2013), which allowed us to estimate the conditional indirect effects by computing confidence intervals using 5000 bootstrap samples.

#### Effects on Body Posture

We first hypothesized that women would mimic the posture of a female, but not a male, role model. To test this hypothesis, we looked at the interaction between role model posture (open vs. closed) and role model gender in predicting participants’ body posture openness. This analysis was identical across all three models, given that the models were different only in terms of the performance outcome. Findings revealed a significant interaction, *b* = .51, *p* = .027. Given that both predictor variables are dichotomous, we probed this interaction by computing planned contrasts. Figure 1 offers a visual representation of this interaction.

As predicted, when primed with a female leader role model displaying an open body posture, female participants showed more open body postures themselves (*M* = 2.44, *SD* = .56) compared to when primed with a female leader role model displaying a closed body posture (*M* = 2.11, *SD* = .56), contrast *t*(41) = 2.01, *p* = .047, *d* = .59. However, when primed with a male role model, women’s body posture openness did not change depending on whether the model was displaying an open (*M* = 2.26, *SD* = .51) or closed (*M* = 2.44, *SD* = .55) body posture, contrast *t*(41) = 1.16, *p* = .25, *d* = .34. In other words, the male model’s posture did not influence female participants’ body posture. These results suggest that women copy the female but not the male, role model posture.

#### Effects on Performance

Our second hypothesis was that changes in participants’ body posture openness as a result of exposure to open role models would subsequently lead to better performance on the public speaking task. Supporting this hypothesis, the more open participants’ body postures, the longer their speeches were (see Table 2a), and the better their speeches were rated by an external coder who watched their speeches (see Table 2b) as well as by an external coder who only listened to their speech (see Table 2c).

**Table 2:** Moderated mediation results for all three outcomes, Study 1

	Coefficient	*p*
(a) Speaking time		
Role Model Posture (Predictor)	−7.97	.52
Role Model Gender (Moderator)		
Participants’ body posture openness (Mediator)	41.92	.001
Role Model Posture x Gender		
IVET realness (Control)	−6.11	.31
Constant	64.33	.05
	*R*^*2*^ = .11 *F* (3, 82) = 3.44, *p* = .02	
(b) External evaluation of speech performance: audio and video		
Role Model Posture (Predictor)	.04	.82
Role Model Gender (Moderator)		
Participants’ body posture openness (Mediator)	.78	.0001
Role Model Posture x Gender		
IVET realness (Control)	−.10	.28
Constant	.76	.14
	*R*^*2*^ = .22 *F* (3, 82) = 7.83, *p* = .0001	
(c) External evaluation of speech performance: audio only		
Role Model Posture (Predictor)	.07	.68
Role Model Gender (Moderator)		
Participants’ body posture openness (Mediator)	.40	.01
Role Model Posture x Gender		
IVET realness (Control)	−.09	.25
Constant	2.17	.0001
	*R*^*2*^ = .11 *F* (3, 82) = 3.44, *p* = .02	

Role Model Posture: 0 = closed; 1 = open; Role Model Gender*:* 0 = male; 1 = female

We also found evidence for moderated mediation for all three outcome variables. First, body posture openness mediated the relation between the role model’s posture and female participants’ speech length, but only when the role model was female (conditional indirect effect *b* = 13.51, *SE* = 8.01, 95% *CI* [.86, 32.88]) and not when the role model was male (conditional indirect effect *b* = −7.82, *SE* = 7.15, 95% *CI* [−25.02, 3.86]). In other words, when exposed to a female, but not to a male, open posture role model, female participants displayed a more open body posture themselves, which in turn led to giving longer speeches.

Similarly, being exposed to a role model with an open body posture led participants to display more postural openness that, in turn, led to better speeches, both when rated based on video with audio and audio only. For video speech performance, as predicted, this mediation was significant only when exposed to a female leader role model (conditional indirect effect *b* = .25, *SE* = .15, 95% *CI* [.009, .59]) and not when exposed to a male role model (conditional indirect effect *b* = −.15, *SE* = .12, 95% *CI* [−.42, .08]). The same pattern of conditional indirect effects was found for speech performance rated based on audio only. More specifically, the mediation was significant when participants were exposed to a female leader role model (conditional indirect effect *b* = .13, *SE* = .08, 95% *CI* [.009, .34]) and not when exposed to a male role model (conditional indirect effect *b* = −.07, *SE* = .07, 95% *CI* [−.25, .03]).

### Discussion

The results of Study 1 show that the experimental manipulation of the female (but not the male) role model’s body posture resulted in changes in the body posture of female participants during a leadership task. Women mimicked the female leader role model’s posture by showing more postural openness when exposed to an open-posture female role model and more postural restriction when exposed to a closed-posture female role model. Importantly, female participants only mimicked the female leader role model and not the male role model, consistent with previous findings showing an ingroup effect (Bourgeois and Hess 2008; van Baaren et al. 2009; Yabar et al. 2006).

Women’s change in postural openness following exposure to a female leader role model explained the effects of the female leader role model on women’s performance as public speakers. The more open participants’ body posture was, the longer women spoke and the better external coders rated their speeches. Taken together, these results show support for the hypothesized empowering mimicry process—female leader role models empowered women’s leadership behavior through increases in postural openness as a result of mimicking the role model.

## Study 2

In our second study we were interested in whether empowering mimicry occurs when women are exposed to any visual representation of a woman during a leadership task or whether the effects are limited to highly successful, known female leader role models. In other words, do female leader role models need to communicate the idea of success in leadership or is exposure to any female exemplar enough to elicit empowering mimicry effects in leadership tasks? We propose two competing hypotheses. If gender is the sole important factor for a model to inspire women (i.e., having any woman represented), we should see similar effects of the female leader role model as we did in Study 1. Women should mimic the female leader role model by showing more open postures when the role model has an open versus closed body posture. Women should also show better performance when the role model has an open versus closed body posture.

However, we argue that the empowering effects of these role models occur in part because women view the female leader as an inspiring role model worth emulating, a hypothesis consistent with the learning goal of mimicry (Hess and Fischer 2017; Kavanagh and Winkielman 2016). Thus, we propose that empowering mimicry effects would not necessarily extend to exposure to non-leader, unknown female targets. These targets will not be categorized as successful leaders and, as a consequence, rather than being perceived as a source of learning, exposure to such targets will likely activate thoughts about an expected social interaction. Furthermore, in such social interactions, responses to dominant behaviors differ from the mimicry responses we demonstrated with role models. More precisely, a complementary response may occur, a response that is common for power-related behaviors in social interactions. Indeed, individuals exposed to confederates displaying open, dominant body postures decreased their own postural openness (Tiedens and Fragale 2003). Because of this prior finding, we propose an alternative hypothesis: that women exposed to an unknown female will display the response most common in social interactions by complementing her body posture. That is, we predict women will show more closed postures when exposed to an open female model compared to a closed female model. The present study is important because it uncovers one of the boundary conditions of female leader role models empowering women in leadership tasks.

### Method

#### Participants and Procedure

We recruited 50 female participants from a Swiss university (*M*_age_ = 22.4-years-old, *SD* = 4.29, range = 18–37). The procedure and measures were identical to Study 1, with participants being asked to give a persuasive political speech in a room designed using IVET. The main difference from Study 1 was that we superimposed on the open and closed body postures the head of an unknown female politician—a local Texas politician with whom Swiss participants were not familiar (see Figure 3s in the online supplement). None of the participants identified the woman during a manipulation check at the end of the experiment.

The behavioral coding was performed similarly as in Study 1. Two trained research assistants coded body posture openness by using the same coding procedure as in the previous study. The reliability between the two coders computed based on the entire sample was excellent, Krippendorff’s *α* = .90, so we averaged their scores (*M* = 1.96, *SD* = .27, range = 1–2.38). In terms of speech performance, we employed the same strategy of two independent coders assessing speech performance based on video. The two coders were sufficiently reliable, Krippendorff’s *α* = .72, so we averaged their scores (*M* = 2.32, *SD* = 1.03, range = 1–5). As in Study 1, all coders were unaware of the experimental condition and the study hypotheses. Given that in Study 1 audio and video coding yielded the same results and were significantly correlated, we no longer performed the audio coding in Study 2. We however assessed speaking time as an objective measure of empowered behavior.

### Results

Table 3 contains means, standard deviations, and correlations for all study variables. Age significantly correlated with one coder’s speech performance evaluation, but analyses with and without this covariate yielded the same results. As such, we report analyses without age as a covariate. IVET realness did not correlate with our outcomes so we did not control for this variable in subsequent analyses. Unlike Study 1, in the present study we recruited participants using a participant pool at a different university. Because of this difference, it is likely that Study 2 participants had more experience with virtual reality, and as a result their feelings of realness did not correlate with outcomes.

**Table 3:** Descriptive statistics and correlations among study variables, Study 2

			Correlations					
Variables	*M*	*SD*	2	3	4	5	6	7
1. Body posture openness 1	1.95	.26	.91**	.28*	.25	.30*	.001	.12
2. Body posture openness 2	1.99	.25	–	.27	.30*	.40**	.05	.26
3. Speaking time	202.20	68.54		–	.65**	.61**	.003	.035
4. Speech performance video 1	2.46	1.03			–	.76**	.16	.16
5. Speech performance video 2	2.19	1.20				–	.34*	.07
6. Age	22.40	4.29					–	−.04
7. IVET realness	1.61	.76						–

*n* = 50. IVET = Immersive Virtual Environment Technology

*p < .05. **p < .01

#### Effects on Body Posture

Women displayed less open body postures when exposed to a model with an open body posture (*M* = 1.89, *SD* = .35) compared to a model with a closed body posture (*M* = 2.04, *SD* = .11), *t(*48) = 2.09, *p* = .042, *d* = .58. In other words, female participants complemented rather than mimicked the unknown woman’s posture.

#### Effects on Performance

Female participants who gave a speech while being exposed to an unknown woman with an open body posture gave shorter speeches (*M* = 178.32, *SD* = 57.88) compared to those who were exposed to an unknown woman with a closed body posture (*M* = 226.08 *SD* = 71.08), *t*(48) = 2.60, *p* = .01, *d* = .74. We obtained similar findings for speech performance as evaluated based on videotapes. Female participants who gave a speech while being exposed to an unknown woman with an open body posture gave speeches that were rated worse (*M* = 1.92, *SD* = .89) compared to those who were exposed to an unknown woman with a closed body posture (*M* = 2.72, *SD* = 1.03), *t*(48) = 2.94, *p* = .005, *d* = .83.

Moreover, using the PROCESS macro, we further investigated whether the effects of role model posture on speech performance and speaking time were due to changes in participants’ body posture. First, participants’ body posture openness was correlated with longer speaking times and with better rated speeches (see Table 3). Second, participants’ body posture mediated the relation between role model posture and both speech performance and speaking time, such that participants exposed to the open posture woman showed more closed body postures, which in turn led to worse performance. The indirect effects calculated with Preacher and Kelley’s (2011) Kappa-squared were significant for both for speech performance (*b* = .07, *SE* = .03, 95% *CI* [.02, .15]) and speaking time as an example of empowered behavior (*b* = .06, *SE* = .04, 95% *CI* [.009, .16]).

### Discussion

Findings show that an unknown female role model does not elicit an empowering mimicry response such that female participants would mimic and be empowered by the open body posture of an unknown female role model. In fact, results suggest the tendency for a complementarity response: Women exposed to an unknown woman with an open posture tended to show less open body postures and lower performance during the leadership task compared to women exposed to an unknown woman with closed posture. These findings are consistent with Tiedens and Fragale’s (2003) complementarity response, a common reaction that serves to maintain smooth functioning via the emergence of an informal hierarchy in social interactions.

These findings suggest that being recognized as a leader is important for a role model to elicit the empowering mimicry response. We can speculate that this is the case because a known successful female leader challenges the negative stereotype of women and serves as an inspiration and a source of learning. As a result, such successful female role models are not seen as possible interaction partners in a certain hierarchy, but rather as icons who inspire and teach women how to behave in challenging situations. An unknown woman, instead, may be seen as a potential interaction partner thus is more likely to elicit a complementarity response.

## General Discussion

The goal of the current studies was to investigate empowering mimicry as a two-step process that explains why visible, successful female role models in leadership empower women in leadership tasks. Findings suggest that women mimicked the body postures of familiar female leader role models by showing more postural openness during speech delivery. Open body postures are an expression of power and dominance so we proposed that adopting these postures as a result of mimicry would lead to better performance on a leadership task, consistent with the literature suggesting an effect of posture on power feelings and behaviors (Carney et al. 2010, 2015; Cuddy et al. 2015). Indeed, women’s postural change translated into more empowered behavior (longer speeches) and better rated performance on the public speaking task.

We also investigated the boundary conditions of the empowering mimicry effect and showed that empowering mimicry does not occur for any female model presented during a task. Specifically, women tended to complement instead of mimic an unknown model, and this postural change led to less empowered behavior and lower speech performance. These findings suggest a boundary condition for this effect: empowerment effects stem from exposure to known, successful leaders. We can speculate that such exposures are successful because they show women how to be and act in challenging situations. Overall, our two studies show that empowering mimicry is an important mechanism through which known female leader role models empower women in leadership, and it occurs when female leader role models are highly visible and clearly successful.

The current research builds on the literature that investigates the relation between postural openness and power-related feelings and behaviors (Carney et al. 2010, 2015; Cuddy et al. 2015). We add to this literature and the controversy surrounding it in two ways. First, we replicate the effects of postural openness on empowered behavior (speaking time) in a context in which participants are faced with a leadership challenge. Second, we show an alternative to artificial power posing. We show that mimicry can, in some situations, be a source of power embodiment. This is a subtle, relatively unconscious process (Chartrand and Bargh 1999) such that participants are not explicitly required to hold certain poses. Although unconscious mimicry effects have been shown in the literature, our findings are theoretically important because they suggest for the first time that these body changes in response to mimicry can actually lead to power embodiment effects. Future work should investigate empirically to what extent effects occurring from mimicry rather than artificial posing are qualitatively or quantitatively different.

Our research also uncovers some of the conditions under which power-related nonverbal behaviors such as body posture elicit complementary versus mimicry effects. Most research so far focused on complementarity reactions, that is, responding to dominance with submission and vice-versa (Tiedens and Fragale 2003). Such complementary reactions are believed to occur because they help maintain social hierarchies and smooth social interactions. However, we show an exception to this pattern. If the target is perceived as an iconic, successful leader, it is likely that a social interaction is not envisioned. Instead, a learning goal may be activated (Hess and Fischer 2017; Kavanagh and Winkielman 2016). As a result, mimicry of the power postures of the recognized leader is more likely to occur.

These findings are an important addition to the female role model literature in leadership because they explain previously mixed findings through mimicry. As we noted before, whereas some studies have shown negative effects of female role models (Hoyt and Simon 2011; Parks-Stamm et al. 2008; Rudman and Phelan 2010), others have shown positive effects (Latu et al. 2013). By showing that empowering mimicry may account for positive effects, we suggest that the opportunity to mimic a visible role model’s nonverbal behavior could thereby partly explain the previously inconsistent effects of female role models for women in leadership. In other words, one factor that influences whether women are inspired or threatened by highly successful female role models is the actual opportunity to mimic these role models. This is not to say that less visible role models cannot inspire women. Instead, we suggest that visibility may increase the chance of inspiring effects because it offers the opportunity for mimicry and nonverbal behavior learning.

### Limitations and Future Directions

The current studies investigated body posture as one power-related behavior that could be mimicked and thus lead to empowering effects. However, it is uncertain if other power-related behaviors would lead to similar empowering mimicry effects. Consequently, future studies should investigate not only different nonverbal behaviors (e.g., visual dominance, voice quality), but also effects across different types of leadership tasks and behaviors. We propose that successful female role models are important because they can change negative gender stereotypes in leadership. However, this is merely speculation, and future studies should investigate whether the behavioral effect that we obtained across our studies is also accompanied by a change in women’s gender-leadership stereotypes such that exposures to powerful female leaders increase the positivity of such stereotypes.

### Practice Implications

From a practice perspective, our findings are important because they show the importance of not only having powerful female leader role models, but also *visible* female role models in leadership. There are many popular articles and books which argue that women should be more visible in leadership (Groysberg and Connolly 2013; Haslam 2015). However, the arguments are often vague and not empirically based. Also, they do not explore the behavioral mechanism through which female leader role models can be empowering. Our studies offer empirical evidence for this claim, as well as an investigation of the mechanism, because we show that continuous visual exposure of the female role models is an important ingredient for inspiring women faced with stressful leadership tasks. This finding would suggest that women’s visibility in leadership should be increased given that it leads to inspiring effects on women with leadership aspirations. In other words, women’s visibility in high power positions should be not only the goal but also a source of women’s advancement in the workplace. Going beyond visibility, our research also suggests that women should be portrayed displaying empowering postures and behaviors.

As a result, practice professionals in schools, universities, and businesses should consider how imagery is used in their environments, both in physical spaces such as classrooms, boardrooms, and conference rooms, as well as in online spaces such as websites and social media accounts. Specifically, practitioners should ensure that women are equally visible in these environments and that images of empowering female role models are brought forward.

### Conclusions

There are numerous moral and pragmatic reasons to increase the number and visibility of competent, successful women in leadership positions. These women serve as powerful role models for women and can have beneficial effects on their behaviors and leadership aspirations. Our research supports this claim and further uncovers the behavioral mechanism that accounts for these positive effects. Our findings show that known, visible female leader role models are vital to inspiring women because they offer the opportunity to mimic their nonverbal behaviors such as powerful body postures. When women adopt these powerful postures themselves, these nonverbal behavioral changes further lead to empowering effects on women’ performance, a process we call empowering mimicry. As a result, our research suggests that increasing the visibility of female leaders can have beneficial effects on women in stressful leadership tasks. These female leader role models can show women how to behave in challenging situations, ultimately serving the goal of empowering women.

## Electronic supplementary material

ESM 1 (DOCX 1570 kb)
