# Supplementary material for: Empowering Mimicry: Female Leader Role Models Empower Women in Leadership Tasks Through Body Posture Mimicry
Source: Sex Roles. 2018 Apr 7;80(1):11–24. doi: 10.1007/s11199-018-0911-y (PMC6318345; doi:10.1007/s11199-018-0911-y)
Supplement: Supplementary file 1 — (DOCX 1570 kb) [file 11199_2018_911_MOESM1_ESM.docx]

Online supplement for Latu, I. M., Schmid, M., Bombari, D., Lammers, J., and Hoyt, C. L. (2018). Empowering mimicry: Female leader role models empower women in leadership through body posture mimicry. *Sex Roles*. Ioana Latu, Queen’s University Belfast. Email: i.latu@qub.ac.uk


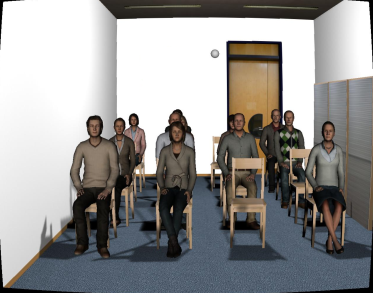


*Figure 1s.* Participants’ perspective while delivering the speech in the virtual reality environment (no role model presented in this picture)


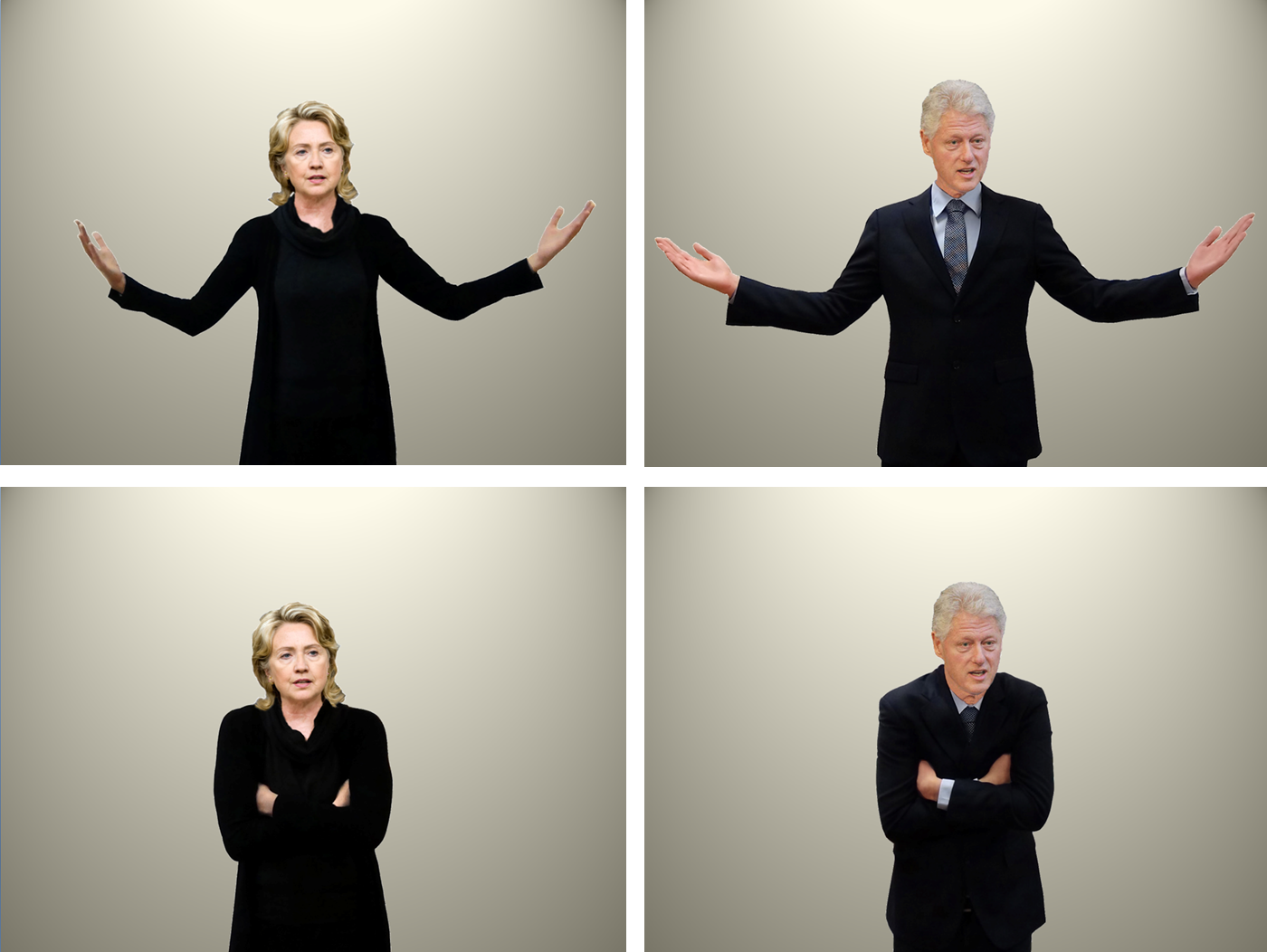


*Figure 2s.* Open (expansive) and closed (restricted) female and male role model pictures for Study 1.


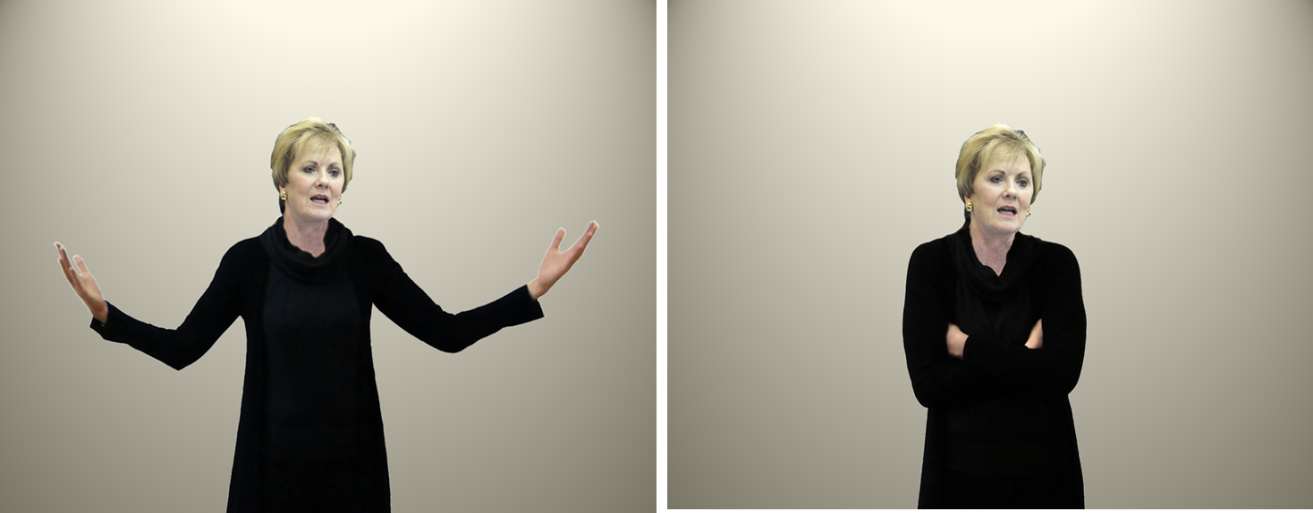


*Figure 3s.* Common model with open and closed body posture in Study 2.
